# Activity of PD-1 Inhibitor Combined With Anti-Angiogenic Therapy in Advanced Sarcoma: A Single-Center Retrospective Analysis

**DOI:** 10.3389/fmolb.2021.747650

**Published:** 2021-11-16

**Authors:** Yang You, Xi Guo, Rongyuan Zhuang, Chenlu Zhang, Zhiming Wang, Feng Shen, Yan Wang, Wenshuai Liu, Yong Zhang, Weiqi Lu, Yingyong Hou, Jing Wang, Xuan Zhang, Minzhi Lu, Yuhong Zhou

**Affiliations:** ^1^ Oncology Department, Zhongshan Hospital, Shanghai, China; ^2^ GenomiCare Biotechnology (Shanghai) Co., Ltd., Shanghai, China

**Keywords:** sarcoma, immunotherapy, anti-angiogenesis, PD-1, biomarker

## Abstract

**Background:** Immune checkpoint inhibitors (ICIs) are employed to treat various cancers, including soft tissue sarcomas (STSs), and less than 20% of patients benefit from this treatment. Vascular endothelial growth factor (VEGF) promotes the immunosuppressive tumor microenvironment and contributes to ICI-resistant therapy. Anti-VEGF receptor tyrosine-kinase inhibitors (TKIs) combined with ICIs have shown antitumor activity in patients with alveolar soft-part sarcoma (ASPS). However, they have not been extensively studied to treat other STS subtypes, such as leiomyosarcoma (LMS), dedifferentiated liposarcoma (DDLPS), undifferentiated pleomorphic sarcoma (UPS), myxofibrosarcoma (MFS), and angiosarcoma (AS).

**Methods:** In this retrospective study, we collected data from 61 patients who were diagnosed with advanced STS based on imaging and histology, including LMS, DDLPS, and UPS. Among them, 41 patients were treated with ICIs combined with TKIs and 20 patients received ICI therapy. The endpoints of progression-free survival (PFS) and overall response rate (ORR) were analyzed in the two groups, and the overall response [partial response (PR), stable disease (SD), and progressive disease (PD)] of each patient was determined using RECIST 1.1 evaluation criteria.

**Results:** In total, 61 STS patients had the following subtypes: LMS (n = 20), DDLPS (n = 17), UPS (n = 8), ASPS (n = 7), MFS (n = 7), and AS (n = 2). The median PFS (mPFS) was significantly prolonged after ICI treatment in combination with TKIs (11.74 months, 95% CI 4.41–14.00) compared to ICI treatment alone (6.81 months, 95% CI 5.43–NA) (HR 0.5464, *p* = 0.043). The 12-month PFS rates of patients who received ICI–TKI treatment were increased from 20.26% (95% CI 0.08–0.53) to 42.90% (95% CI 0.27–0.68). In the combination therapy group, 12 patients (30%) achieved PR, 25 patients (62.5%) achieved SD, and 3 patients (7.5%) achieved PD for 3 months or longer. In the non-TKI-combination group, 2 patients (9.5%) achieved PR, 14 patients (66.7%) achieved SD, and 5 patients (23.8%) achieved PD within 3 months. The ORRs in the two groups were 30.0% (ICI–TKI combination) and 9.5% (ICI only), respectively. A notable ORR was observed in the ICI–TKI combination group, especially for subtypes ASPS (66.7%), MFS (42.9%), and UPS (33.3%). The PD-L1 expression (n = 33) and tumor mutation burden (TMB, n = 27) were determined for each patient. However, our results showed no significant difference in PFS or response rates between the two groups.

**Conclusion:** This study suggests that ICI–TKI treatment has antitumor activity in patients with STS, particularly the ASPS and MFS subtypes. Moreover, effective biomarkers to predict clinical outcomes are urgently needed after combination therapy in the STS subtypes.

## Introduction

Sarcoma represents a heterogeneous group of various soft tissue and bone tumors of mesenchymal origin that accounts for approximately 1% of adult malignancies and 15% of pediatric malignancies and is comprised of over 100 different subtypes (Fletcher et al., 2013). Although soft tissue sarcoma (STS) is a rare cancer, the global annual incidence of STS is 1.8–5.0/100,000 in people younger than 45 years (Siegel et al., 2021). Surgery (combined with chemotherapy and radiotherapy) remains the mainstay treatment for local STS, but up to 40% of patients experience tumor recurrence and inevitably progress to advanced disease (Judson et al., 2014). For advanced STS, anthracycline-based chemotherapy or other drug combinations have been widely used albeit with limited benefit, and with restricted success when utilized as second-line or systemic therapies (Savina et al., 2017). Therefore, more effective agents and therapeutic strategies need to be explored for STS treatment.

Immune checkpoint inhibitors (ICIs; anti-PD-1/PD-L1 and anti-CTLA4 antibodies) are a compelling new option for the treatment of various advanced cancers, including sarcomas. Previous studies have shown that the expression of PD-1/PD-L1 in sarcoma patients has a strong positive correlation with T cell infiltration and B cell activation (Kim et al., 2021; Petitprez et al., 2020). In the SARC028 and Alliance A0914401 clinical trials, anti-PD-1 agents (pembrolizumab and nivolumab) or those combined with an anti-CTLA agent (ipilimumab) have shown clinical benefits in advanced/metastatic sarcoma, but the overall response rate (ORR) in all cohorts was only 18 and 16%, respectively (D’Angelo et al., 2018; Tawbi et al., 2017). Most non-responders have a significant correlation with restricted infiltration of immune cells, such as T cells and macrophages, and PD-L1 expression levels. The immunosuppressive tumor microenvironment (TME) generated by the dysfunctional tumor immune system leads to resistance to immunotherapy (Rabinovich et al., 2007). It has been proved that angiogenesis contributed to the maintenance of immunosuppressive TME in renal cell carcinoma and melanoma and was associated with antitumor activity (Yang et al., 2018); however, its roles and related mechanisms for immunosuppressive actions in sarcoma remain unexplored.

Vascular endothelial growth factor (VEGF) is considered to be the main driver gene of angiogenesis, leading to tumor growth and metastasis, and it also contributes to suppression of the immunotherapy response (Fukumura et al., 2018). Therefore, the anti-VEGF receptor tyrosine-kinase inhibitors (TKIs) display anti-cancer activity against STS, including anlotinib, pazopanib, and regorafenib (Berry et al., 2017; Chi et al., 2018; Weiss et al., 2020). Notably, on the basis of the role of VEGF in the suppressive TME, immune checkpoint inhibitor–based therapies combined with TKIs have exhibited favorable outcomes in various types of cancers (Fukumura et al., 2018). According to a phase 3 trial, axitinib plus pembrolizumab exhibited anti-cancer activity in patients with ASPS (Wilky et al., 2019). However, the safety and efficiency of immunotherapy combined with TKIs in other sarcoma subtypes are still to be determined.

In this retrospective study, we collected data from 61 STS patients treated with ICIs or ICIs combined with TKIs. The aims were to determine the progression-free survival (PFS) and overall response rate (ORR) for five sarcoma subtypes and to correlate the patients’ demographics and clinical data that may predict improved outcomes.

## Methods

### Patients and Study Design

In this single-institution retrospective cohort study, we selected 61 STS patients diagnosed with STS. The subjects of this study were patients with sarcoma at any stage who received ICI (PD-1/PD-L1 inhibitor) or ICI–TKI treatment at Zhongshan Hospital Affiliated to Fudan University, China, from January 1, 2015, to April 10, 2021. The ICI agents were administered to patients with STS, including LMS, DDLPS, ASPS, UPS, MFS, and AS; the TKI agents include anlotinib, pazopanib, and regorafenib. The exclusion criteria for patients were 1) autoimmune disease, 2) rheumatic disease, or 3) active bleeding. By reviewing their electronic medical records, the treatment history, demographics, genetics, pathology, and radiology information were retrospectively collected. If ≥1% of various markers in the collected tumor tissues were stained positive, it was considered that the expression of PD-L1 in the tumor was positive. The study protocol was approved by the Ethics Committee of Zhongshan hospital (IRB protocol number B2020-338).

### Immunohistochemistry (IHC)

The PD-L1 expression in each patient was determined by immunohistochemistry. The tumor tissue slides were baked at 63°C for 60 min and then boiled in 10 mM sodium citrate (pH 6.0) for 30 min to retrieve the antigen. Anti-PD-L1 polyclonal antibody was used as the primary antibody (Abcam, Cambridge, United Kingdom; rabbit SP142, 1:300; Dako IHC 22C3, 1:300) and goat anti-rabbit biotinylated IgG as the secondary antibody (Maxim, UltraSensitive^TM^ SP IHC Kit, KIT-9707). The slides were then counterstained with hematoxylin (Gene, GT100540), dehydrated, and mounted. Images were digitally scanned at ×20 magnification.

### Statistical Analysis

A radiologist certified by the board of directors performed tumor measurements on the lesions found on the patient’s CT or MRI imaging and evaluated the clinical response of the patients to ICI treatment according to Response Evaluation Criteria in Solid Tumors Version 1.1 (RECIST 1.1). In order to include patients in the PFS analysis, they were required to have a baseline scan within 3 weeks of the first ICI treatment. In addition, follow-up scans were required at least 9 weeks after the start of ICI treatment. The best tumor response was evaluated using the RECIST 1.1 standard and defined as the maximum reduction in the target tumor burden. Kaplan–Meier analysis was used to compare PFS rates between the two treatment groups. To perform PFS analysis on patients who were lost to follow-up or survived without PFS at the end of the study, the data were checked at the last tumor imaging. All statistical analyses were performed using GraphPad Prism (GraphPad Software, CA, United States).

## Results

### Patient Demographics

Sixty-one patients were included in the study with various subtypes, including leiomyosarcoma (LMS, n = 20), dedifferentiated liposarcoma (DDLPS, n = 17), undifferentiated pleomorphic sarcoma (UPS, n = 8), ASPS (n = 7), myxofibrosarcoma (MFS, n = 7), and angiosarcoma (AS, n = 2). Among these patients, males accounted for 54.1%, the median age was 48.5 years, and all of them were Asians (Supplementary Table S1). Among them, 21 patients received ICI treatment only and 40 patients received ICIs combined with TKIs. The baseline characteristics between the two groups have no significant difference and are listed in Table 1. At the beginning of ICI treatment, 58 patients had metastatic disease (95.1%) and 3 patients had local disease (4.9%). The most common tumor sites were the retroperitoneum (n = 49), followed by the uterus (n = 7), heart (n = 2), paranasal sinus (n = 1), and mediastinum (n = 1). Most of the patients had already undergone chemotherapy and received a median of two cycles of conventional treatment (range 1–4) before ICI or ICI–TKI treatment. The main common reasons interrupting the treatment were progression of the disease or intolerable toxicity (Supplementary Table S2).

**TABLE 1:** Baseline characteristics of patients with STS.

	ICIs (n = 21)	ICIs + TKIs (n = 40)	Total (n = 61)	*p*-Value
Gender	—	—	—	0.227
Female	12	22	1/20	—
Male	9	18	5%	—
Age (average, years)	48	48	4/17	—
Primary tumor location	—	—	—	0.377
Retroperitoneum	15	24	39	—
Heart	0	2	2	—
Uterus	2	5	7	—
Extremities	2	5	7	—
Others	2	4	6	—
Chemo treatment	—	—	—	0.485
Gemcitabine	5	14	19	—
Anthracyclines	5	10	15	—
Dacarbazine	2	2	4	—
Best response	—	—	—	0.071
PR	2	12	14	—
SD	14	25	39	—
PD	5	3	8	—
Immunotherapy	—	—	—	0.297
PD-1 inhibitor	21	38	58	—
PD-L1 inhibitor	0	2	2	—
PD-L1 status	—	—	—	0.114
Positive	8	8	16	—
Negative	4	13	17	—
FNCLCC	—	—	—	0.356
II	4	12	16	v
III	17	28	45	
Histological grade	—	—	—	0.262
Grade II	4	4	8	—
Grade III	9	22	31	—
Unknown	8	14	22	—

### Efficacy

**FIGURE 1:**
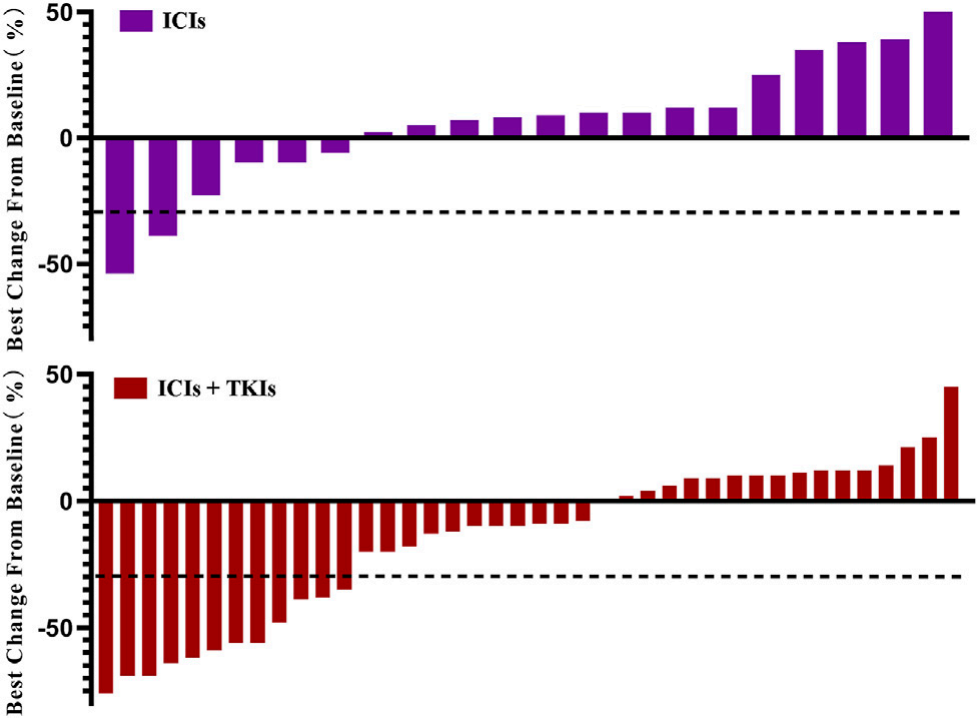
Change of tumor burden from baseline. Patients who received immune checkpoint inhibitors (ICIs; n = 21) or ICIs combined with tyrosine-kinase inhibitors (TKIs; n = 40) are listed. Each bar represents one patient.

Of the 61 subjects, 21 received ICI treatment (group A) and 40 received ICI–TKI combination treatment (group B; Table 2). Patients’ response to treatment was assessed be measuring the tumor size according to the RECIST 1.1 criteria (Figure 1). In group A, 2 patients achieved a partial response (PR), 14 achieved stable disease (SD), and 5 had progressive disease (PD). In group B, 12 patients achieved a partial response (PR), 25 achieved stable disease (SD), and 3 had progressive disease (PD). Thus, the ORR in groups A and B was 9.5 and 30.0%, respectively. Although no patients achieved complete response (CR), one DDLPS patient achieved pCR after surgery, one patient had a pathological response rate up to 86%, and three patients had a pathological PR [at least 30% reduction in the target tumor mutation burden (TMB)].

**TABLE 2:** Treatment strategies in STS subtypes.

	ICIs (n = 21)	ICIs + TKIs (n = 40)	Total (n = 60)	*p*-Value
LMS (n = 20)	0/5	1/15	1/20	0.62
	0%	6.7%	5%	—
DDLPS (n = 17)	0/6	4/11	4/17	0.09
	0%	36.3%	23.5%	
UPS (n = 8)	2/5	1/3	3/8	0.85
	40%	33.3%	37.5%	—
ASPS (n = 7)	0/4	2/3	2/7	0.05
	0%	66.7%	28.6%	—
MFS (n = 7)	0	3/7	3/7	—
	—	42.9%	42.9%	—
AS (n = 2)	0	1/2	1/2	—
	—	50%	50%	—

**FIGURE 2:**
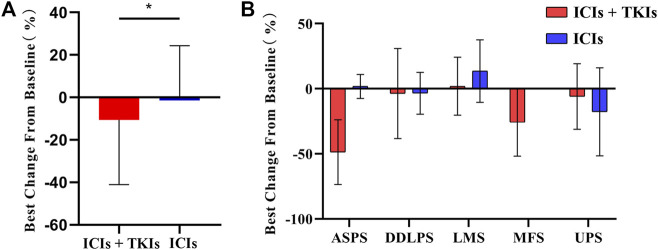
Clinical response rate in soft tissue sarcoma (STS) subtypes: **(A)** tumor burden changes from baseline in the two groups; **(B)** changes of tumor burden in STS subtypes. ASPS, alveolar soft-part sarcoma; DDLPS, dedifferentiated liposarcoma; LMS, leiomyosarcoma; MFS, myxofibrosarcoma; UPS, undifferentiated pleomorphic sarcoma.

Considering the best response rate in less than 3 months, PR, SD, and PD accounted for 24.6, 60.7, and 14.8%, respectively (Table 3). Compared to ICI treatment alone, ICI–TKI treatment enhanced the ORR and DCR (CR + PR + SD) in the first 3 months (ORR 10 vs. 31.7%; DCR 80 vs. 87.9%; Figure 2A). Moreover, we found that seven patients received mono-immunotherapy, four ASPS (2 PR and 2 SD for best response), one LMS (SD for 3 months and PD for 6 months), one UPS (SD for 1 year), and one DDLPS (PD for 4 months). Regarding the different STS subtypes, there was a significant difference in the response between the two groups who received immune-based treatment. In general, UPS, ASPS, and MFS patients had the highest response rates, with DDLPS greatly fluctuating and LMS having a low response rate (Figure 2B). In addition, we also found that patients with ASPS were sensitive to ICI–TKI combination treatment. However, those with UPS appeared to benefit from ICIs instead of the combination treatment. All seven patients with MFS received ICI–TKI combination treatment, and they responded satisfactorily. DDLPS appeared to maintain SD, regardless of whether TKI treatment was used or not, with regression of the lesion not observed. Although LMS patients had the lowest response rate in both groups, the patients achieved rapid progress in the ICI treatment group compared to the combination treatment group (Supplementary Figure S1).

**TABLE 3:** Clinical response rate of soft tissue sarcoma subtypes in the two groups.

	ICIs (n = 21)	ICIs + TKIs (n = 40)	Total (n = 61)	*p*-Value
PR	2/21	12/40	14/61	0.07
	9.5%	30.0%	23.0%	
SD	14/21	25/40	39/61	0.75
	66.7%	62.5%	63.9%	
PD	5/21	3/40	8/61	0.07
	23.8%	7.5%	13.1%	
mPFS	7 (2–19)	12 (2–NA)	9 (2–NA)	—

**FIGURE 3:**
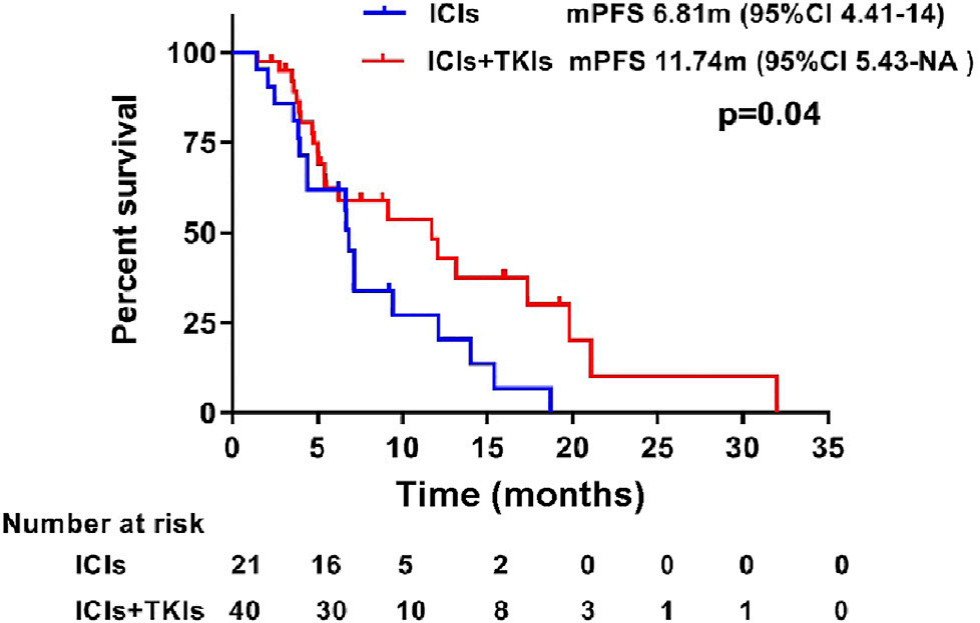
Progression-free survival between treatment with immune checkpoint inhibitors (ICIs) and ICIs combined with tyrosine-kinase inhibitors (TKIs). Kaplan–Meier estimates showed a significant difference in progression-free survival in patients treated with ICIs and ICIs combined with TKIs.

Progression-free survival (PFS) during treatment of the two groups was also assessed. Our results showed that the median PFS (mPFS) was significantly prolonged in the ICI–TKI group (11.74 months) compared to the ICI group (6.81 months; HR 0.55, 95% CI 0.2673–0.9791, *p* = 0.043; Figure 3). The 12-month PFS rate for patients who were given ICIs in combination with TKIs was increased from 20.26% (95% CI 0.078–0.53) to 42.9% (95% CI 0.27–0.68).

**FIGURE 4:**
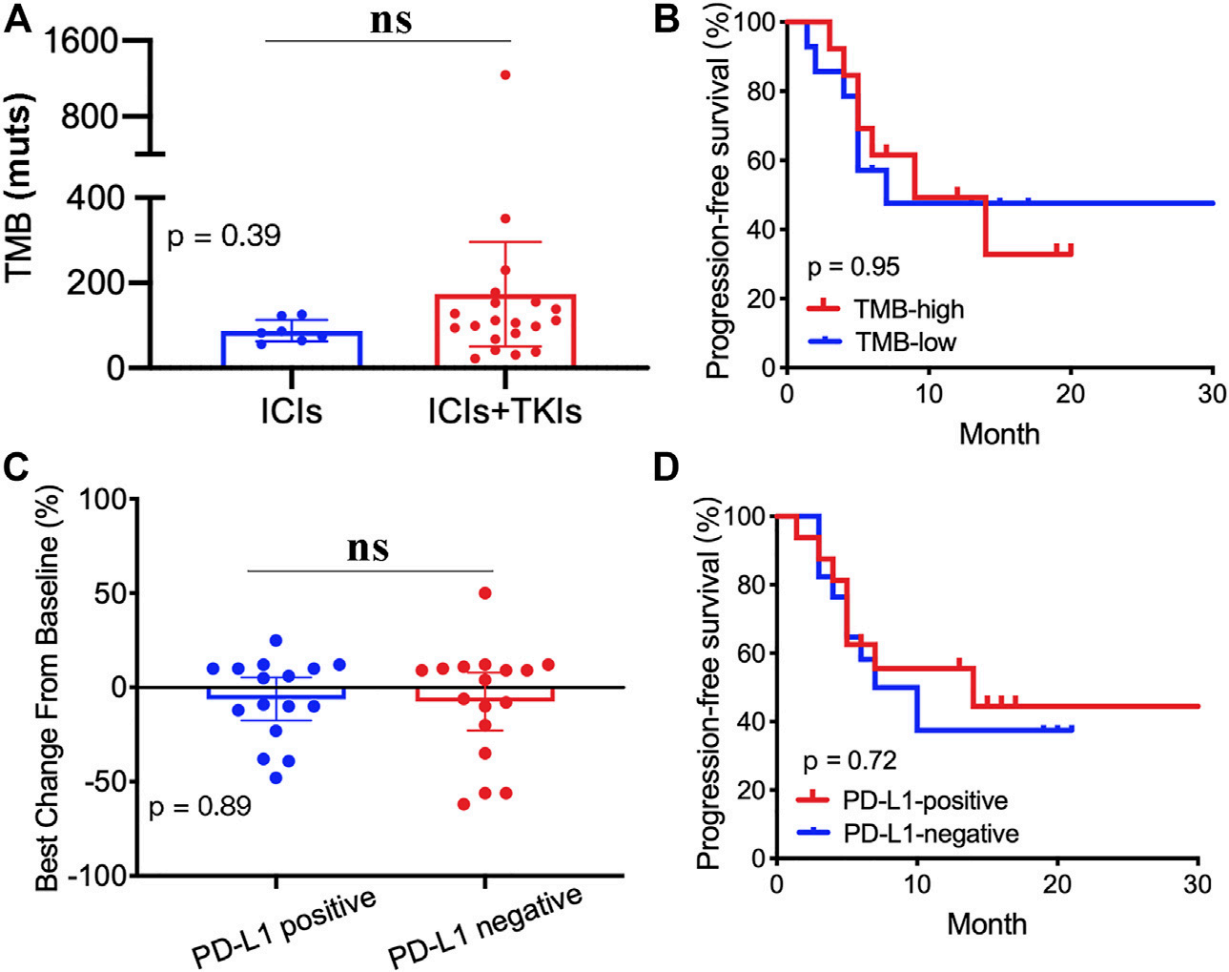
Correlation between tumor mutation burden (TMB), PD-L1 expression, and clinical outcomes: **(A)** TMB value in the two groups; **(B)** Kaplan–Meier analyses based on the TMB value; **(C)** tumor mutation burden changes from baseline between PD-L1–positive and PD-L1–negative groups; **(D)** Kaplan–Meier analyses based on the PD-L1 status.

In order to explore the correlation between TMB and treatment, we carried out next-generation sequencing in 27 patients who received ICI (8/27) or ICI–TKI (19/27) treatment. Our results showed no significant differences in the TMB value between the two groups (Figure 4A). Moreover, the patients were divided into two groups based on the TMB value (TMB-high and TMB-low). We also found that the PFS was not significantly different (Figure 4B). We supposed that the expression of PD-L1 could predict the clinical benefit of ICI–TKI treatment. Our results revealed that there was no difference in the rate of change of tumor (Figure 4C) and in the PFS (Figure 4D) in the two treated groups. Overall, our results demonstrated that both the TMB and the PD-L1 status were not satisfactory predictors to distinguish clinical outcomes.

### Safety

Treatment-related adverse events, as graded by Common Terminology Criteria for Adverse Events (CTCAE) Version 4.0, included rash (14.8%), fever (16.4%), fatigue (26.2%), hypothyroidism (18%), hypertension (50.8%), elevated ALT/AST/ALP levels (41.0%), vitamin D deficiency (37.7%), hyperlipidemia (34.4%), anemia (23.0%), and dental ulcer (2/61, 3.3%). None grade 4 CTCAE occurred in either groups (Supplementary Table S3).

## Discussion

Emerging data suggest that immunotherapy has become a new therapeutic model in oncology. However, the results of clinical trials have shown that the majority of STS patients do not benefit from this treatment (total response rate <20%) (Monga et al., 2020). Tumor angiogenesis not only contributes to tumor growth and metastasis but also induces the immunosuppressive formation of TME (Yi et al., 2019). Therefore, the combination of immunotherapy and anti-angiogenic therapy has recently emerged as a novel treatment pattern (Fukumura et al., 2018; Wang et al., 2020; Zhang et al., 2019). Based on the single-arm clinical trial of NCT02636725, the anti-VEGF inhibitor (axitinib) plus anti-PD-1 antibody (pembrolizumab) had antitumor activity in ASPS patients (the mPFS was 12.4 months, the ORR was 54.5%, and the CBR was 72.7% at 3 months) (Wilky et al., 2019). However, data on the real-world clinical effectiveness of combination therapy in ASPS and other STS subtypes remain scarce.

Sixty-one eligible patients with various STS subtypes were included in this real-world study. Twenty-one patients received immunotherapy, and 40 patients received immunotherapy in combination with anti-angiogenesis inhibitors. Our results showed that combination therapy exerts a good therapeutic effect, providing evidence for the effectiveness of this treatment pattern in STS (Figure 1). Although the mixture of low- and high-grade sarcomas makes comparison difficult, we have made certain observations in this heterogeneous sarcoma and therapy combination.

First, patients with ASPS had the best response to immunotherapy, although with limited sample size (7 patients). Among them, about 71.4% exhibited a strong partial response (Supplementary Figure S2). Two of the patients who received combination therapy achieved SD for more than 3 months, far beyond than expected (Tawbi et al., 2017). In addition, we found that the response rate was higher in the combination therapy group than in the ICI therapy group (10 vs. 31.7%). These findings were consistent with the results of a prospective phase 2 clinical trial, where the patients with ASPS who received axitinib in combination with pembrolizumab exhibited significant improvements in mPFS, ORR, and CBR (Wilky et al., 2019). We also showed that the combination of immunotherapy and anti-vascular therapy has advantages for the treatment of ASPS subtypes, and we are looking forward to more data and mechanism research to further illustrate this point of view.

Previous studies have shown that LMS subtypes respond to immunotherapy, with an ORR of 45% (Tawbi et al., 2017). However, in our cohort, 37.5% (6/16) of LMS patients achieved PD and 50% (8/16) achieved SD. Monga et al. (2020) suggested that the TMB alteration of multiple lines of chemotherapy or radiation in the real world could lead to different response rates for immunotherapy treatment. We did not investigate TMB or PD-L1 status within the patients, which is a limitation in our study. Although LMS patients in our study did not benefit from either ICI or ICI–TKI treatment, we found that the patients achieved SD instead of PD in the combination therapy group (Figure 2). It is therefore important to investigate the efficacy and safety of combination therapy for LMS in future randomized clinical trials and to discover prognostic biomarkers that may assist in response prediction. In addition, we verified the effectiveness of PD-1 inhibitors for UPS, but in the process by boldly trying anti-vascular TKI combination. We found that the combination did not further improve the efficacy of these patients in terms of PFS and ORR. Our results suggest that UPS, as a complex and heterogeneous STS, requires further exploration of a more appropriate combination plan to screen patients who will benefit.

MFS is similar to UPS in the analysis of genotypes, and both were considered to be the same responding subtype to ICI for its TIL microenvironment (Pollack et al., 2017). Seven MFS patients in our cohort were given combination therapy as a leading exploration. Our results showed that the patients not only had a satisfactory ORR (42.9%) but also prolonged the PFS, and all patients achieved SD. Our center actively evaluated four patients: two received ICI–TKI two-drug combination therapy and two received ICI combined with chemotherapy and TKI three-drug combination therapy, and the results are encouraging. We found that the ORR reached 50% and CBR reached 100%. This result gives us great confidence to further perform the larger-scale, mechanism research of immunotherapy-combined anti-vascular targeted therapy in MFS.

Previous studies have demonstrated that PD-L1 expression is a prognostic factor for STS immunotherapy (Kim et al., 2021). However, we found that the status of PD-L1 did not predict the CBR of ICI–TKI treatment. Moreover, the TMB was not a satisfactory marker to predict clinical outcomes in STS patients who received immune-based therapies.

The tolerability of immunotherapy in sarcoma patients appears to be similar to that in other patients (Horvat et al., 2015). Rash, fever, and fatigue were the most common adverse events. Similar to other experiences with immunotherapeutic agents, certain unusual toxicities were observed, necessitating discontinuation of the drug and administration of steroids (D’Angelo et al., 2018).

## Conclusion

In general, anti-angiogenesis inhibitors combined with PD-1 inhibitor therapy can enhance the mPFS (more than 5 months), ORR, and DCR in patients with STS. Our study showed that patients with ASPS and UPS are more sensitive to immunotherapy and that MFS is another promising subtype to benefit from ICI–TKI combination therapy. Although LMS does not significantly change the ORR of immunotherapy, we found that the mPFS of LMS patients who received immunotherapy was significantly prolonged.

## Data Availability

The original contributions presented in the study are included in the article/Supplementary Material, and further inquiries can be directed to the corresponding author.

## References

[B1] Berry V. Basson L. Bogart E. Mir O. Blay J.-Y. Italiano A. (2017). REGOSARC: Regorafenib versus Placebo in Doxorubicin-Refractory Soft-Tissue Sarcoma-A Quality-Adjusted Time without Symptoms of Progression or Toxicity Analysis. Cancer 123, 2294–2302. 10.1002/cncr.30661 28295221 PMC5485075

[B2] Chi Y. Fang Z. Hong X. Yao Y. Sun P. Wang G. (2018). Safety and Efficacy of Anlotinib, a Multikinase Angiogenesis Inhibitor, in Patients with Refractory Metastatic Soft-Tissue Sarcoma. Clin. Cancer Res. 24, 5233–5238. 10.1158/1078-0432.CCR-17-3766 29895706

[B3] D’Angelo S. P. Mahoney M. R. Van Tine B. A. Atkins J. Milhem M. M. Jahagirdar B. N. (2018). A Non-comparative Multi-center Randomized Phase II Study of Nivolumab +/− Ipilimumab for Patients with Metastatic Sarcoma (Alliance A091401). Lancet Oncol. 19, 416–426. 10.1016/S1470-2045(18)30006-8 29370992 PMC6126546

[B4] Fletcher C. Bridge J. Hogendoorn P. Mertens F. (2013). WHO Classification of Tumours of Soft Tissue and Bone. 4th edition (Wold Health Organization, Lund University Publishtions).

[B5] Fukumura D. Kloepper J. Amoozgar Z. Duda D. G. Jain R. K. (2018). Enhancing Cancer Immunotherapy Using Antiangiogenics: Opportunities and Challenges. Nat. Rev. Clin. Oncol. 15, 325–340. 10.1038/nrclinonc.2018.29 29508855 PMC5921900

[B6] Horvat T. Z. Adel N. G. Dang T.-O. Momtaz P. Postow M. A. Callahan M. K. (2015). Immune-Related Adverse Events, Need for Systemic Immunosuppression, and Effects on Survival and Time to Treatment Failure in Patients with Melanoma Treated with Ipilimumab at Memorial Sloan Kettering Cancer Center. J. Clin. Oncol. 33, 3193–3198. 10.1200/JCO.2015.60.8448 26282644 PMC5087335

[B7] Judson I. Verweij J. Gelderblom H. Hartmann J. T. Schöffski P. Blay J.-Y. (2014). Doxorubicin Alone versus Intensified Doxorubicin Plus Ifosfamide for First-Line Treatment of Advanced or Metastatic Soft-Tissue Sarcoma: a Randomised Controlled Phase 3 Trial. Lancet Oncol. 15, 415–423. 10.1016/S1470-2045(14)70063-4 24618336

[B8] Kim S. K. Kim J. H. Kim S. H. Lee Y. H. Han J. W. Baek W. (2021). PD-L1 Tumour Expression Is Predictive of Pazopanib Response in Soft Tissue Sarcoma. BMC Cancer 21, 336. 10.1186/s12885-021-08069-z 33789622 PMC8011221

[B9] Monga V. Skubitz K. M. Maliske S. Mott S. L. Dietz H. Hirbe A. C. (2020). A Retrospective Analysis of the Efficacy of Immunotherapy in Metastatic Soft-Tissue Sarcomas. Cancers 12, 1873. 10.3390/cancers12071873 PMC7408640 32664595

[B10] Petitprez F. de Reyniès A. Keung E. Z. Chen T. W.-W. Sun C.-M. Calderaro J. (2020). B Cells Are Associated with Survival and Immunotherapy Response in Sarcoma. Nature 577, 556–560. 10.1038/s41586-019-1906-8 31942077

[B11] Pollack S. M. He Q. Yearley J. H. Emerson R. Vignali M. Zhang Y. (2017). T-cell Infiltration and Clonality Correlate with Programmed Cell Death Protein 1 and Programmed Death-Ligand 1 Expression in Patients with Soft Tissue Sarcomas. Cancer 123, 3291–3304. 10.1002/cncr.30726 28463396 PMC5568958

[B12] Rabinovich G. A. Gabrilovich D. Sotomayor E. M. (2007). Immunosuppressive Strategies that Are Mediated by Tumor Cells. Annu. Rev. Immunol. 25, 267–296. 10.1146/annurev.immunol.25.022106.141609 17134371 PMC2895922

[B13] Savina M. Le Cesne A. Blay J.-Y. Ray-Coquard I. Mir O. Toulmonde M. (2017). Patterns of Care and Outcomes of Patients with METAstatic Soft Tissue SARComa in a Real-Life Setting: the METASARC Observational Study. BMC Med. 15, 78. 10.1186/s12916-017-0831-7 28391775 PMC5385590

[B14] Siegel R. L. Miller K. D. Fuchs H. E. Jemal A. (2021). Cancer Statistics, 2021. CA A. Cancer J. Clin. 71, 7–33. 10.3322/caac.21654 33433946

[B15] Tawbi H. A. Burgess M. Bolejack V. Van Tine B. A. Schuetze S. M. Hu J. (2017). Pembrolizumab in Advanced Soft-Tissue Sarcoma and Bone Sarcoma (SARC028): a Multicentre, Two-Cohort, Single-Arm, Open-Label, Phase 2 Trial. Lancet Oncol. 18, 1493–1501. 10.1016/s1470-2045(17)30624-1 28988646 PMC7939029

[B16] Wang Q. Gao J. Di W. Wu X. (2020). Anti-angiogenesis Therapy Overcomes the Innate Resistance to PD-1/pd-L1 Blockade in VEGFA-Overexpressed Mouse Tumor Models. Cancer Immunol. Immunother. 69, 1781–1799. 10.1007/s00262-020-02576-x 32347357 PMC11027722

[B17] Weiss A. R. Chen Y.-L. Scharschmidt T. J. Chi Y.-Y. Tian J. Black J. O. (2020). Pathological Response in Children and Adults with Large Unresected Intermediate-Grade or High-Grade Soft Tissue Sarcoma Receiving Preoperative Chemoradiotherapy with or without Pazopanib (ARST1321): a Multicentre, Randomised, Open-Label, Phase 2 Trial. Lancet Oncol. 21, 1110–1122. 10.1016/S1470-2045(20)30325-9 32702309 PMC7745646

[B18] Wilky B. A. Trucco M. M. Subhawong T. K. Florou V. Park W. Kwon D. (2019). Axitinib Plus Pembrolizumab in Patients with Advanced Sarcomas Including Alveolar Soft-Part Sarcoma: a single-centre, Single-Arm, Phase 2 Trial. Lancet Oncol. 20, 837–848. 10.1016/S1470-2045(19)30153-6 31078463

[B19] Yang J. Yan J. Liu B. (2018). Targeting VEGF/VEGFR to Modulate Antitumor Immunity. Front. Immunol. 9, 978. 10.3389/fimmu.2018.00978 29774034 PMC5943566

[B20] Yi M. Jiao D. Qin S. Chu Q. Wu K. Li A. (2019). Synergistic Effect of Immune Checkpoint Blockade and Anti-angiogenesis in Cancer Treatment. Mol. Cancer 18, 60. 10.1186/s12943-019-0974-6 30925919 PMC6441150

[B21] Zhang G. Liu C. Bai H. Cao G. Cui R. Zhang Z. (2019). Combinatorial Therapy of Immune Checkpoint and Cancer Pathways Provides a Novel Perspective on Ovarian Cancer Treatment (Review). Oncol. Lett. 17, 2583. 10.3892/ol.2019.9902 30854033 PMC6365948

