# Supplementary figures and images for: Activity of PD-1 Inhibitor Combined With Anti-Angiogenic Therapy in Advanced Sarcoma: A Single-Center Retrospective Analysis
Source: Front Mol Biosci. 2021 Nov 16;8:747650. doi: 10.3389/fmolb.2021.747650 (PMC8635153; doi:10.3389/fmolb.2021.747650)

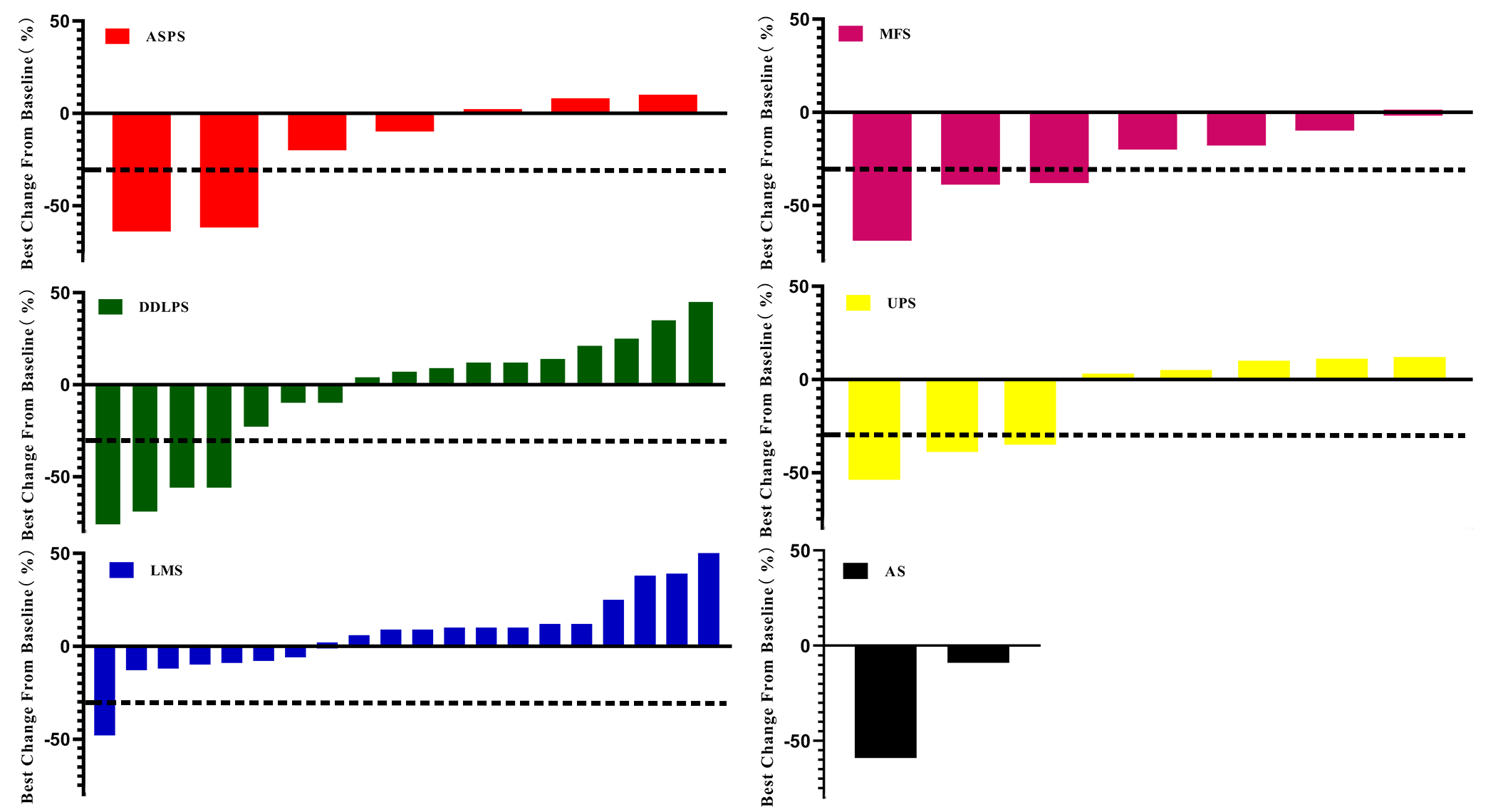

Supplement: Supplementary file 1 [file Image2.TIF]

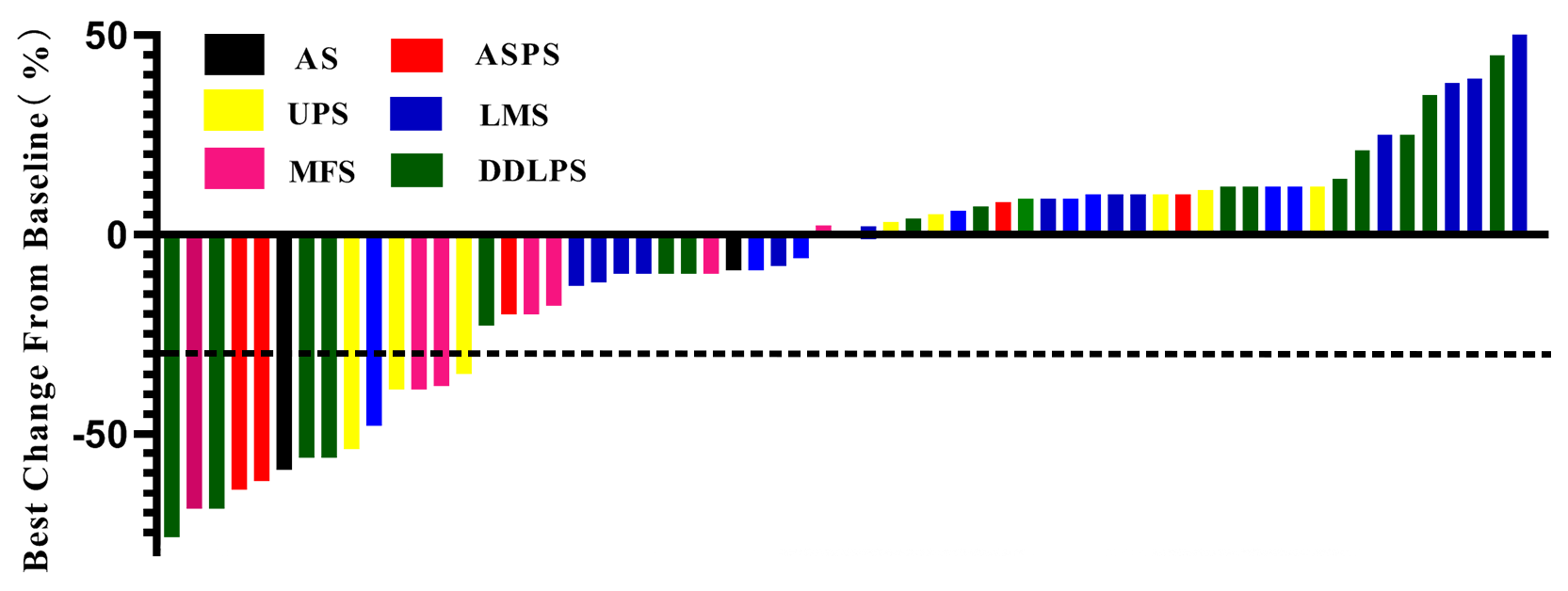

Supplement: Supplementary file 2 [file Image1.TIF]
