# Supplementary material for: Activity of PD-1 Inhibitor Combined With Anti-Angiogenic Therapy in Advanced Sarcoma: A Single-Center Retrospective Analysis
Source: Front Mol Biosci. 2021 Nov 16;8:747650. doi: 10.3389/fmolb.2021.747650 (PMC8635153; doi:10.3389/fmolb.2021.747650)
Supplement: Supplementary file 3 [file Table2.docx]

**Table S2 Baseline characteristics of STS subtypes**

|  | | **LMS** | **DDLPS** | **UPS** | **ASPS** | **MFS** | **AS** | **Total** |
| --- | --- | --- | --- | --- | --- | --- | --- | --- |
|  |  | **20（32.8%）** | **17（27.9%）** | **8（13.1%）** | **7（11.5%）** | **7（11.5%）** | **2（3.3%）** | **61** |
| **Gender** | **Female** | **14** | **7** | **5** | **3** | **3** | **1** | **33（54.1%）** |
|  | **Male** | **6** | **10** | **3** | **4** | **4** | **1** | **28（45.9%）** |
| **Age at diagnosis** | **Average** | **49** | **54** | **55** | **31** | **44** | **48** | **53** |
| **Primary Tumor**  **Location** | **Retroperitoneal** | **13** | **17** | **4** | **0** | **5** | **0** | **39（63.9%）** |
|  | **Uterus** | **7** | **0** | **0** | **0** | **0** | **0** | **7（11.5%）** |
|  | **Extremities** | **0** | **0** | **0** | **7** | **0** | **0** | **7（11.5%）** |
|  | **Other** | **0** | **0** | **1** | **0** | **2** | **2** | **5（1%）** |
| **Chemo treatment** | **Gemcitabine** | **6** | **5** | **3** | **0** | **2** | **2** | **18（29.5%）** |
|  | **Anthracyclines** | **3** | **8** | **1** | **0** | **3** | **0** | **15（24.6%）** |
|  | **Dacarbazine** | **2** | **1** | **0** | **0** | **1** | **0** | **4（6.6%）** |
| **Immunotherapy**  **treatment** | **PD-1 inhibitor** | **19** | **7** | **8** | **7** | **6** | **2** | **59（96.7%）** |
|  | **PD-L1 inhibitor** | **1** | **0** | **0** | **0** | **1** | **0** | **2（32.8%）** |
| **TKI treatment** | **TKI addition** | **15** | **11** | **2** | **3** | **7** | **2** | **40（65.6%）** |
|  | **Without TKI** | **5** | **6** | **6** | **4** | **0** | **0** | **21（34.4%）** |
| **Number of prior lines of treatment** | **Median** | **2** | **1** | **2** | **1** | **2** | **1.5** | **2** |
|  | **Range** | **1-4** | **1-3** | **1-2** | **1-2** | **1-2** | **1-2** | **1-4** |
| **PD-L1 status** | **Positive** | **8** | **5** | **3** | **0** | **2** | **0** | **18** |
|  | **Negative** | **7** | **3** | **3** | **1** | **2** | **0** | **16** |
| **FNCLCC** | **II** | **9** | **5** | **2** | **2** | **2** | **0** | **20(32.8%)** |
|  | **III** | **11** | **12** | **6** | **5** | **5** | **2** | **41(67.2%)** |
| **Histological grade** | **2** | **4** | **/** | **1** | **2** | **1** | **/** | **8(13.1%)** |
|  | **3** | **9** | **9** | **5** | **2** | **5** | **1** | **31(50.8%)** |
|  | **Not applicable** |  |  |  |  |  |  | **22(36.1%)** |
