# Supplementary material for: Activity of PD-1 Inhibitor Combined With Anti-Angiogenic Therapy in Advanced Sarcoma: A Single-Center Retrospective Analysis
Source: Front Mol Biosci. 2021 Nov 16;8:747650. doi: 10.3389/fmolb.2021.747650 (PMC8635153; doi:10.3389/fmolb.2021.747650)
Supplement: Supplementary file 4 [file Table3.docx]

**Table S3 Treatment-related adverse events**

|  | **ICI（N=21）** | | **ICI+TKI（N=40）** | | **Total （N=61）** |
| --- | --- | --- | --- | --- | --- |
|  | **G1-2** | **G3** | **G1-2** | **G3** |  |
| **Rash** | **3（14.3%）** | **/** | **5（12.5%）** | **1（2.5%）** | **9(14.8%)** |
| **Hypothyroidism** | **3（14.3%）** | **/** | **8（20%）** | **/** | **11(18.0%)** |
| **Hypertension** | **5（23.8%）** | **2（9.5%）** | **18（45%）** | **6（15%）** | **31(50.8%)** |
| **Fever** | **3（14.3%）** | **1（4.8%）** | **6（15%）** | **/** | **10(16.4%)** |
| **Fatigue** | **5（23.8%）** | **/** | **9（22.5%）** | **2（5%）** | **16(26.2%)** |
| **Dental Ulcer** | **1（4.8%）** | **/** | **/** | **1（2.5%）** | **2(3.3%)** |
| **Elevated ALT, AST, or AP** | **7（33.3%）** | **/** | **15（37.5%）** | **2（5%）** | **24(39.3%)** |
| **Seizures** | **1（4.8%）** | **/** | **/** | **/** | **1(1.6%)** |
| **Headache** | **/** | **/** | **2（5%）** | **/** | **2(3.3%)** |
| **Haemoptysis** | **/** | **/** | **3(7.5%)** | **/** | **3(4.9%)** |
| **Anemia** | **3（14.3%）** | **1（4.8%）** | **9（22.5%）** | **1（2.5%）** | **14(23.0%)** |
| **Arthralgia or myalgia** | **3（14.3%）** | **/** | **5（12.5%）** | **/** | **8(13.1%)** |
| **BUN increased** | **1（4.8%）** | **/** | **4（10%）** | **1（2.5%）** | **6(9.8%)** |
| **Proteinuria** | **1（4.8%）** | **1(1.6%)** | **5（12.5%）** | **/** | **7(11.5%)** |
| **Epistaxis** | **1（4.8%）** | **/** | **1（2.5%）** | **/** | **2(3.3%)** |
| **Pneumothorax** | **1（4.8%）** | **/** | **/** | **/** | **1(1.6%)** |
| **Hyperglycaemia** | **4（19.0%）** | **/** | **5（12.5%）** | **1（2.5%）** | **10(16.4%)** |
| **Vitamin D deficiency** | **7（33.3%）** | **1（4.8%）** | **10（25%）** | **5（12.5%）** | **23(37.7%)** |
| **cTNT elevation** | **2（9.5%）** | **/** | **4（10%）** | **/** | **6(9.8%)** |
| **Hyperlipemia** | **10（47.6%）** | **/** | **11（27.5%）** | **/** | **21(34.4%)** |
